# Adeno-associated virus type 2 preferentially integrates single genome copies with defined breakpoints

**DOI:** 10.1186/1743-422X-11-15

**Published:** 2014-01-27

**Authors:** Tyler Janovitz, Michel Sadelain, Erik Falck-Pedersen

**Affiliations:** 1 Tri-Institutional MD-PhD Program, Weill Medical College of Cornell University, New York, NY 10065, USA; 2 Department of Microbiology and Immunology, Weill Medical College of Cornell University, New York, NY 10065, USA; 3 Memorial Sloan-Kettering Cancer Center, New York, NY 10065, USA

**Keywords:** AAV-2, Integration, Virus chromosomal junctions, Viral breakpoints

## Abstract

**Background:**

Adeno-associated virus (AAV) serotype 2 prevalently infects humans and is the only described eukaryotic virus that integrates site-preferentially. In a recent high throughput study, the genome wide distribution of AAV-2 integrants was determined using Integrant Capture Sequencing (IC-Seq). Additional insight regarding the integration of AAV-2 into human genomic DNA could be gleaned by low-throughput sequencing of complete viral-chromosomal junctions.

**Findings:**

In this study, 140 clones derived from Integrant-Capture Sequencing were sequenced. 100 met sequence inclusion criteria, and of these 39 contained validated junction sequences. These unique sequences were analyzed to investigate the structure and location of viral-chromosomal junctions.

**Conclusions:**

Overall the low-throughput analysis confirmed the genome wide distribution profile gathered through the IC-Seq analysis. We found no unidentifiable sequence inserted at AAV-2 chromosomal junctions. Assessing both left and right ends of the AAV genome, viral breakpoints predominantly occurred in one hairpin of the inverted terminal repeat and AAV genomes were preferentially integrated as single copies.

## Findings

Adeno-associated virus, a human Parvovirus in the genus *Dependovirus*, possesses a linear single-strand 4.7 Kb genome [1]. AAV serotype 2 infects up to eighty percent of the human population [2,3] and is the only described eukaryotic virus that integrates site-preferentially [4-6]. The dominant integration hotspot, AAVS1, is located in the first exon of protein phosphatase 1 regulatory subunit 12C (PPP1R12C) [1,7]. Site-preferential integration requires two cell-extrinsic factors: the large AAV replication proteins, Rep68 or Rep78 [8-11], and DNA integration substrates containing Rep binding sites, which are GAGC repeats [12-14].

The genome-wide integration profile of AAV-2 has recently been revealed by a high-throughput sequencing approach coupled with bioinformatics [15]. That study was the first high-throughput analysis of AAV integration and led to a number of discoveries, including the presence of several thousand novel genomic hotspots. However, paired-end sequencing generates short reads that do not sequence the entirety of viral-chromosomal junctions.

**Figure 1:**
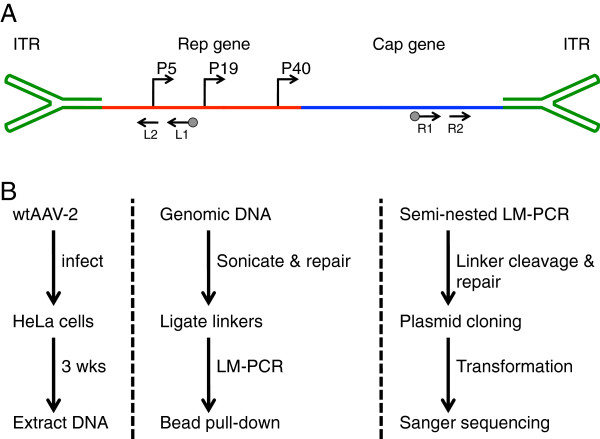
**Schematic of AAV genome and experimental design. (A)** Overview of AAV genome features (elements of this diagram are not to scale). Inverted terminal repeats (green) cap the ends of the single-strand 4.7 Kb viral genome. Expression of two viral genes, Rep (red) and Cap (blue), is driven by three promoters, P5, P19, P40. L1/L2 and R1/R2 (black arrows) are locations for left and right primer pair binding sites, with biotinylation indicated (grey circle). **(B)** Modified IC-Seq outline for junction analysis. AAV-2 infected HeLa cells were grown for three weeks prior to DNA extraction. Human genomic DNA was sonicated, blunted, A-tailed, and ligated to T-tailed asymmetric linkers. Integration junctions were amplified by semi-nested ligation-mediated PCR, incorporating bead pull-down target enrichment. Libraries were then cloned into bacterial plasmids, transformed, and sequenced.

We reasoned that additional insight regarding the integration of AAV-2 into human genomic DNA could be gleaned by low-throughput sequencing of complete viral-chromosomal junctions. In this study, junctions were assayed from wild-type AAV-2 infected HeLa cells processed through the Integrant-Capture Sequencing (IC-Seq) protocol (Figure 1A and B). AAV-2 generated from helper-free plasmid transfection (Applied Viromics) and applied at 1E4 viral genomes per cell. These conditions provide maximal integration efficiency with minimal residual episomal virus, as previously described [15,16]. Since this protocol generates random chromosomal breaks using sonication and does not rely on locus-specific primers, it should be less biased than previous junction studies [15,17,18]. Primer sets to both the left and right portions of the AAV-2 genome were used to assess each biological replicate. The L1/L2 primer set was previously described [15,19] and the R1/R2 oligonucleotides were five-prime modified from a previous study [20] to include [Bio-TEG]C and CGTTT respectively. Methods for IC-Seq are presented in references [15,17,18], and we hope to publish a step-by-step methods guide for future reference. Subsequent to the main phase of IC-Seq, sample pools were cloned into bacterial plasmids, and individual clones were sequenced.

**Figure 2:**
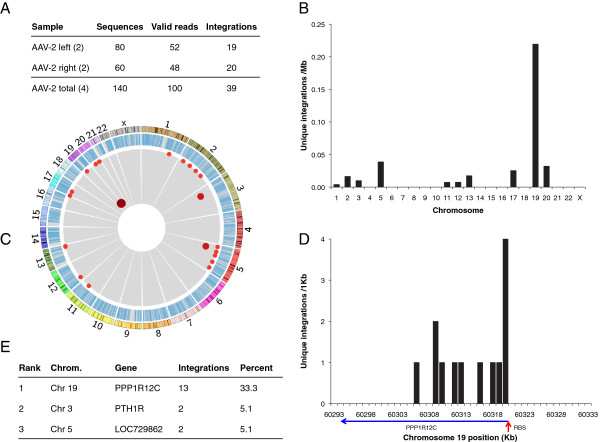
**Chromosomal distribution of integration junctions. (A)** Summary of junction data obtained using both the left and right AAV-2 primer sets; the number in parenthesis represents biological replicates. **(B)** Unique integration events per mappable megabase of human chromosomes. **(C)** Genome-wide view of all integration events (red dots) and genes (blue bars). Darkness, size, and proximity to the center correspond with increasing insertions per locus. Chromosome sizes and banding patterns are presented in the outermost ring. **(D)** Profile of unique integrations around AAVS1 in 1 Kb intervals, with genes and gene orientation (blue arrow). RBS = Rep binding site of AAVS1 (red arrow). **(E)** Summary of most frequent AAV integration loci; all sites with greater than one insertion are shown.

In total, 140 clones were sequenced encompassing two biological replicates assayed using both primer sets (Figure 2A). One hundred of the clones met the inclusion criteria for valid sequences, as previously described [15]. Briefly, these criteria included the presence of correct AAV sequence following the AAV-specific primer and correct linker-tag sequence on the opposing end. These sequence constraints mitigate the possibility of artifactual products [15,17,18]. Of the 100 validated sequences, 39 contained AAV-chromosomal junctions. The remaining sequences were relatively short, representing either uninterrupted viral genome, or viral sequence with a DNA fragment too small to unambiguously assign to the human genome. The 39 confirmed cellular sequences captured averaged 103 base pairs, allowing high-confidence placement in the human genome. Since both the location of the integration junction in the viral inverted terminal repeats and the nature of cellular sequences recovered for the left and right primer sets were extremely similar, they were pooled for further analysis.

Integration junction sequences mapped to ten chromosomes, with chromosome 19 receiving 36% of all events (Figure 2B). Three genomic loci were represented by greater than one unique integrant (Figure 2C and E). AAVS1 was the most frequent site of viral genome insertion, accounting for one-third of all events, while the other two sites, PTH1R (chromosome 3) and LOC729862 (chromosome 5), each represented five percent of detected integrations. These were also the three largest hotspots identified via IC-Seq [15], and two of these hotspots were detected in a previous low-throughput analysis [19]. The thirteen unique integrants identified in AAVS1 begin proximal to the AAV Rep binding site and span the first 15 Kb of PPP1R12C (Figure 2D). This distribution mirrors, on a diminutive scale, the peak-and-tail integration phenotype described in the high-throughput analysis [15].

**Figure 3:**
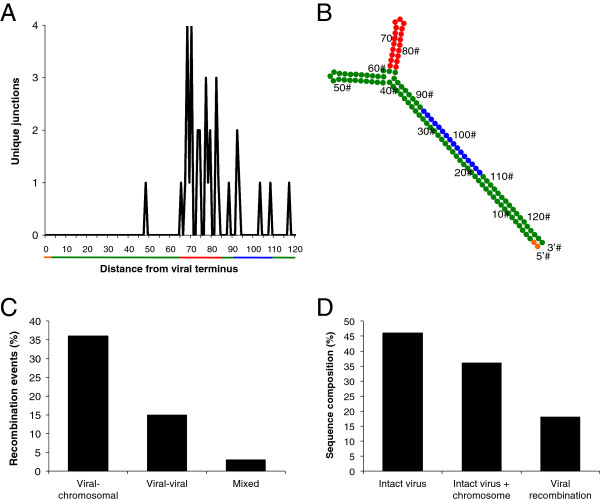
**ITR recombination frequency and structure. (A)** Number of unique viral breakpoints recorded for each nucleotide position in the first 120 bases of the ITR; numeral represents distance from the viral 5*'* end. Colored bars correlate with ITR features as described in panel **B**. **(B)** Nucleotide positions and features of the AAV ITR, with numerals indicating distance from 5*'* viral end. Green represents unassigned activity; red represents the recombination hotspot hairpin; blue denotes the rep binding sites; and orange reflects the Rep nicking site (TRS). **(C)** The percent of total recombination events involving viral-chromosomal junctions, viral-viral junctions, and mixed junctions, i.e. both viral-viral and viral-chromosomal recombination in the same molecule. **(D)** The percent of total validated sequences that are intact virus, viral-chromosomal junctions with intact, single-copy virus, and sequences involving viral-viral recombination.

Examining the viral portion of the recombination junctions revealed additional insights into AAV-2 integration biology. Of the 39 integrations, 92.3% involved contiguous, identifiable viral sequence ligated directly to human chromosomal sequence. The three instances that displayed non-contiguous viral sequence involved viral-viral recombination events in addition to the viral-chromosomal recombination. For the sequences in which contiguous viral regions recombined with chromosomal DNA, 91.6% of viral junctions occurred in the external 120 bp of the inverted terminal repeats (Figure 3A). Within this region, a 19 bp span (position 65-83) accounts for 69.4% of all viral junctions. This small sequence forms one of the two hairpin loops of the viral ITR and is part of the region that occurs in either a flip or flop orientation [21]. Its position relative to the Rep binding and nicking sites provides insight that may explain the targeting of this region (Figure 3B). After a Rep complex engages the Rep binding sequence (RBS), the terminal resolution site (TRS) is nicked, and the complex proceeds with 3′-5′ helicase activity [22-24]. Therefore, the hairpin loop identified as a viral recombination hotspot is the first strong secondary structure encountered by the amplification polymerase complex and may serve to halt progression long enough to facilitate recombination with host DNA via cellular pathways. Additionally, previous work has identified that this internal hairpin loop is specifically bound by AAV Rep during ITR nicking [25]. Thus, the positioning of the Rep nicking complex may contribute to the creation of the observed recombination hotspot.

Several previous studies, mostly involving AAV vectors, have identified the ITRs as frequent viral recombination points in the absence of Rep [20,21,26,27]. Since the AAV genome is linear and flanked by ITRs, viral-cellular recombination would be expected to occur in this region. Additionally, the complex secondary structure of the ITRs is sufficient to induce a host DNA damage response [28-30]. Based on the data presented in this study, and considering the accumulated insight from previous work [20,21,26-30], the identification of the extreme targeting of one specific ITR hairpin as the primary recombination hotspot is an important observation.

Interestingly, the data provided in this study offer insight into the question of whether wild-type AAV genomes integrate as single copies or concatamers. Previous work using Southern blotting to characterize integrations from several cell lines suggested that AAV integrates as head-to-tail concatamers [31]. The data analyzed in this study are one hundred unique sequences from a diverse cell population. Of the one hundred sequences that met our inclusion criteria, forty-six were intact viral sequence, thirty-six were direct viral-chromosomal events, fifteen were viral-viral recombinations and three sequences possessed both viral-viral and viral-chromosomal recombination. Therefore, 66.7% of all recombination events captured were between single viral genomes and human chromosomal DNA (Figure 3C). Additionally, we noted that 82% of all sequences were free of viral-viral recombinations (Figure 3D). Thus, analyzing both ends of integrated AAV-2 sequences, the data indicate viral genomes predominantly integrate into host DNA as single copies.

This study of complete viral-chromosomal junctions derived from cloning and sequencing IC-Seq DNA pools provides valuable insight into AAV integration. The structurally complex, repetitive, and GC-rich nature of these sequences may hinder capture of the entire junction-population. We have taken many steps to mitigate these effects. These steps included using: short sequences from random breaks, two primer sets, stringent sequence validation, robust polymerases, and high melting temperatures. Therefore, we believe that the junctions captured and analyzed in this study are not unduly influenced by sequence constraints, and present a valuable representation of the AAV-2 junction population. The insertion profile of AAV-2 maintained the same top three hotspots found using high-throughput technology and the distribution around AAVS1, the largest hotspot, was also quite similar. In the absence of Rep, the unique AAV-2 ITR structure is a target for cellular DNA repair and recombination pathways which can vary in a cell dependent manner [21,30,32,33]. In the case of wild-type AAV-2, Rep binding to the RBE as well as the hairpin stem influences helicase activity [25]. Therefore, Rep, in concert with cellular DNA repair complexes, may contribute to formation of the internal stem-loop ITR recombination hotspot identified in this study. We anticipate that cell-specific differences in DNA repair proteins and Rep interacting proteins may also influence the integration profile to some extent. However, direct Rep-DNA interactions appear to play the dominant role in defining the genome-wide targets for AAV-2 integration [15,19]. Finally, based on the population of junctions captured, AAV-2 genomes were found to predominately integrate as single genome copies, and viral-viral recombination was modest. This study may impact Rep-mediated gene therapy approaches and highlights how long read length, even on a modest scale, may serve to significantly augment the understanding of high-throughput data sets.

## Competing interests

The authors declare that they have no competing interests.

## Authors’ contributions

TJ designed and performed experiments and analysis and wrote the manuscript. MS provided material assistance and made suggestions on the manuscript. EFP designed experiments and analysis and wrote the manuscript. All authors read and approved the final manuscript.
